# Cognitive capacity in amyotrophic lateral sclerosis: the value of diagnostic markers in cerebrospinal fluid and the influence of nutrition and pulmonary function

**DOI:** 10.1093/braincomms/fcaf137

**Published:** 2025-04-08

**Authors:** Sabrina M Wölfel, Catherine N Widmann, Sergio Castro-Gomez, Patrick Weydt, Pawel Tacik, Michael T Heneka

**Affiliations:** Center for Neurology, Department of Parkinson, Sleep and Movement Disorders, University Hospital Bonn, Bonn 53127, Germany; Center for Neurology, Department of Parkinson, Sleep and Movement Disorders, University Hospital Bonn, Bonn 53127, Germany; Center for Neurology, Department of Parkinson, Sleep and Movement Disorders, University Hospital Bonn, Bonn 53127, Germany; Institute of Physiology II, University Hospital Bonn, Bonn 53115, Germany; Center for Neurology, Department of Parkinson, Sleep and Movement Disorders, University Hospital Bonn, Bonn 53127, Germany; German Center for Neurodegenerative Diseases (DZNE), Bonn 53127, Germany; Center for Neurology, Department for Neuromuscular Disorders, University Hospital Bonn, Germany, Bonn 53127, Germany; Center for Neurology, Department of Parkinson, Sleep and Movement Disorders, University Hospital Bonn, Bonn 53127, Germany; Luxembourg Centre for Systems Biomedicine (LCSB), University of Luxembourg, Belvaux 4367, Luxembourg

**Keywords:** amyotrophic lateral sclerosis, cognition, cerebrospinal fluid, nutrition, pulmonary function

## Abstract

Amyotrophic lateral sclerosis is an incurable neurodegenerative disease that is fatal with a median of 3–4 years. It is characterized by degeneration of the first and second motor neurons. In addition to physical limitations, neuropsychological abnormalities occur in more than 50% of cases. This leads to a rapid loss of autonomy and increases the need for care. An individual prognosis for the course of the disease, in particular the development of cognitive and behavioural abnormalities, is not yet possible As part of our investigations, we focused on cognitive performance and behavioural abnormalities measured by the Edinburgh Cognitive and Behavioural ALS Screen in patients with amyotrophic lateral sclerosis and investigated possible prognostic biomarkers in cerebrospinal fluid as well as modifiable factors such as nutrition and lung function. A retrospective data analysis of 99 patients with amyotrophic lateral sclerosis cases examined between 2018 and 2021 at the Department for Neurodegenerative Diseases and Gerontopsychiatry at the University Hospital of Bonn, using Edinburgh Cognitive and Behavioural ALS Screen, revealed that elevated levels of total tau and phospho-tau 181 were associated with diminished performance of patients with amyotrophic lateral sclerosis on the Edinburgh Cognitive and Behavioural ALS Screen. Additionally, weight loss during the course of the disease has been observed to have a deleterious impact on cognitive performance. Moreover, we were able to demonstrate a previously insufficiently described correlation between abnormalities in the Edinburgh Cognitive and Behavioural ALS Screen and low-normal thiamine levels in serum. The hypothesis that reduced lung function has a negative effect on cognitive performance was not supported by our findings. The initial onset of amyotrophic lateral sclerosis, whether bulbar or spinal, does not appear to affect cognition and behaviour measured using Edinburgh Cognitive and Behavioural ALS Screen. Furthermore, our findings confirm the utility of the Edinburgh Cognitive and Behavioural ALS Screen in identifying a behavioural variant frontotemporal dementia in amyotrophic lateral sclerosis patients who have been previously diagnosed by experienced neurologists using the Rascovsky criteria. This development facilitates a more precise utilization of complex diagnostic instruments. Our results provide insight into the prognosis of patients with amyotrophic lateral sclerosis in terms of cognitive performance and behavioural abnormalities as the disease progresses, as well as potential therapeutic approaches to stabilize and support neuropsychological abnormalities. The importance of total tau as a widely available prognostic marker should be emphasized. Additionally, new avenues of research are emerging, particularly regarding the role of thiamine in amyotrophic lateral sclerosis.

## Teaser

Retrospective data analysis of 99 patients with amyotrophic lateral sclerosis to evaluate prognostic biomarkers and modifiable factors related to cognition and behavioural abnormalities. The continued relevance of total tau in neurodegenerative diseases should be reemphasized. A surprising potential therapeutic target—A high normal thiamine level as a positive prognostic marker.

## Graphical Abstract

**Graphical Abstract:**
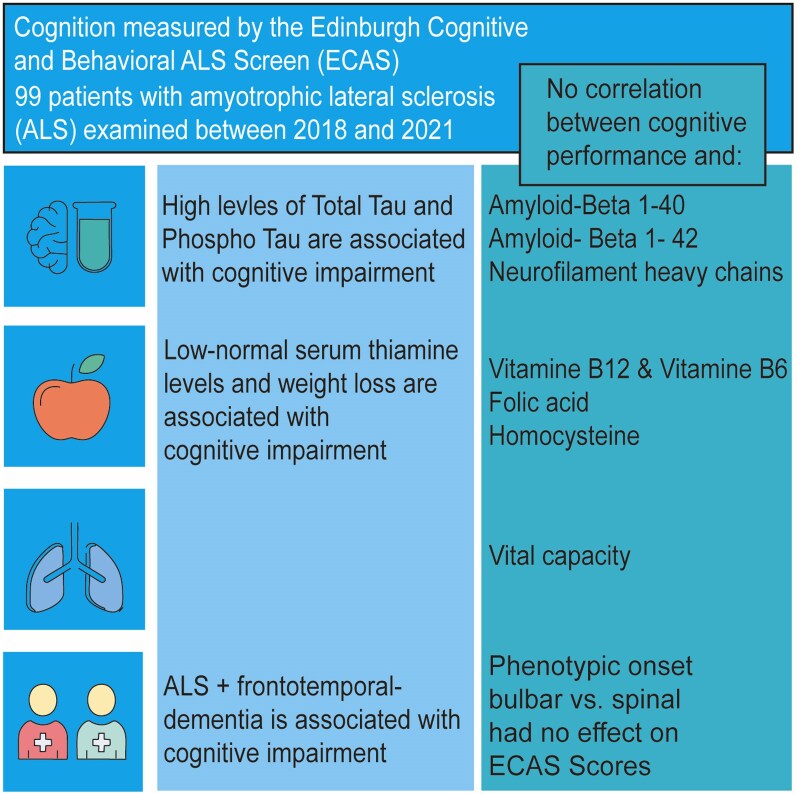

## Introduction

Amyotrophic lateral sclerosis is known as an incurable neurodegenerative disease that leads to progressive signs of paralysis and muscle atrophy, dysarthria, dysphagia and respiratory insufficiency with fatal outcomes due to degeneration of the first and second motor neurons. The average disease duration is 3–4 years.^1^ In addition to physical limitations, cognitive decline and behavioural abnormalities are common during the course of the disease.^2^ In 2013, Abrahams *et al*. developed the Edinburgh Cognitive and Behavioural ALS Screen (ECAS) to capture and quantify these changes.^3,4^ The ECAS consists of 15 individual tasks and assesses amyotrophic lateral sclerosis-specific (language, fluency and executive function) and amyotrophic lateral sclerosis-non-specific domains (memory and spatial perception). In addition to the physical limitations that already lead to a loss of autonomy in patients, this is significantly exacerbated by cognitive limitations and behavioural abnormalities. This is a burden for both patients and caregivers. It is not yet possible to predict the individual course of the disease. Intensive research is currently focusing on the identification of prognostic biomarkers.^5-8^

The treatment of amyotrophic lateral sclerosis consists mainly of symptomatic management of distressing symptoms. In addition, riluzole and edavarone have been shown to have a disease-delaying effect.^9,10^ Other substances from the class of antisense oligonucleotides offer hope for a modification of the disease in patients with a genetic form of amyotrophic lateral sclerosis.^11^ There are also data on the beneficial effects of the use of various micronutrients, particularly vitamin B12.^12^

Almost all publications use the ALS Functional Rating Scale-Revised (ALSFRS-R) as a reference marker for the assessment of disease progression^13^ with the drug or the biomarker under investigation. Only, a few publications have addressed the influence or predictability of cognitive performance.

This led us to perform retrospective data analysis to find prognostic biomarkers with a focus on correlation with cognitive performance. We looked at neurodegeneration markers from cerebrospinal fluid, including neurofilament heavy chains, indicative markers of malnutrition and disease-related ventilatory impairment.

## Materials and methods

A retrospective data analysis was performed on a total of 99 patients (51 females, 48 males) with amyotrophic lateral sclerosis (pALS), who were examined between 2018 and 2021 using ECAS at the Department for Neurodegenerative Diseases and Gerontopsychiatry at the University Hospital of Bonn. The mean age of our population was 66.3 years (*n* = 99; range: 42.0–86.9 years) with a standard deviation of 8.9 years. The mean disease duration at presentation was 1.9 years (*n* = 95; range: 0.1–10.7 years) with a standard deviation of 1.9 years, and the mean ALSFRS-R was 34.55 points (*n* = 83; range: 14–48) with a standard deviation of 8.29 (Table 1). Our cohort had an average educational level of 13.2 years of schooling plus years of study (range: 7–21 years), with a standard deviation of 3.3 years. So, the population was very heterogeneous. The diagnosis of amyotrophic lateral sclerosis was made by experienced neurologists on the basis of the revised El Escorial criteria.^14^ Neuropsychological examination and assessment using the German version of the ECAS was carried out by an experienced team of neuropsychologists. The ECAS was usually administered once and shortly after initial presentation to our clinic but thus at different stages of the disease. Bulbar onset was documented in 18 of these patients, and spinal onset in 79. In the remaining patients, the onset of the disease could not be clearly determined from the medical records. Medical records showed that 6 patients met the Rascovsky *et al.*^15^ criteria for behavioural variant frontotemporal dementia and were diagnosed as such by a neurologist.

**Table 1:** Epidemiological data

Epidemiological data	
Gender	
Male	48
Female	51
Average duration of illness, *n* = 95 (years)	
Average value	1.9
Standard deviation	1.9
ALSFRS-R, *n* = 83	
Average value	34.55
Standard deviation	8.29

In 73 of the above-mentioned patients, lumbar puncture with determination of neurodegeneration-associated proteins was performed as part of an inpatient symptom assessment. Neurodegeneration-associated proteins including amyloid beta 1–40, amyloid beta 1–42 (norm >600 pg/ml), total tau (tTau, norm <400 pg/ml) and phospho-tau 181 (pTau, norm <65 pg/ml) were determined. A lumbar puncture with the determination of neurofilament heavy chain (norm <560 pg/ml) was performed in 75 patients. A blood test for folic acid (norm 4.5–32.2 ng/ml) was performed in 52 of the patients. Vitamin B1 (norm 66.1–200.6 nmol/l) and vitamin B6 (norm 8.7–27.2 µg/l) levels were determined in 50 patients. Vitamin B12 levels (norm 200–1000 pg/ml) were measured in 52 patients and homocysteine levels (norm 3.2–10.7 µmol/l) in 49 patients. In 76 of the patients, height and weight before the onset of the disease and (anamnestic) weight at the time of presentation to our clinic were recorded in close temporal relation to the time of neuropsychological examination so that body mass index (BMI) before and during the course of the disease could be determined and the difference calculated. Expiratory vital capacity (VC) was measured using pulmonary function testing in 68 patients. We used this parameter to evaluate lung function.

Within the framework of the regulations on self-research by service providers [Section 6 of the German Health Data Utilization Act (GDNG)], we did not require explicit patient consent for our retrospective data analysis of the data obtained as part of standard care. We have received confirmation from the Ethics Committee of the University Hospital of Bonn that our evaluations do not require an ethics vote.

### Statistical analysis

Data were collected and analysed using Excel, SPSS and GraphPad Prism. We used the Kolmogorov-Smirnov test to test our variables for normal distribution. Except for average weight loss, our variables were found to be normally distributed. To eliminate the impact of outliers, we excluded one patient from our analyses. This patient had particularly poor results in the ECAS. Given our sample size, we carried out the correlations using Pearson’s correlation coefficient.^16^ We calculated the significance level (*t*) and the corresponding *P*-value. We set the significance level at 0.05. Receiver operating characteristic (ROC) and area under the curve (AUC) analyses were used to determine possible cut-off values. We used a one-way ANOVA with a fixed significance level of 5% to compare different groups within our total population.

## Results

Of the 99 pALS examined by ECAS, 40 patients had a pathological ECAS total score, with a mean score of 104.3 (maximum score 136, cut score 105) and a standard deviation of 18.1 points. In the amyotrophic lateral sclerosis-specific score, 38 pALS showed a pathological result. The mean score was 76.6 (maximum score 100, cut score 77) with a standard deviation of 13.6 points. The average achieved score of our analysed cohort in the amyotrophic lateral sclerosis-non-specific score was 27.7 points (maximum achievable score 36, cut score 24). The standard deviation was 6.2 points. Nineteen pALS had a pathological result in the amyotrophic lateral sclerosis-non-specific score. Contrary to previous research findings,^17^ we demonstrated a diminishing negative effect of age on ECAS total score results (*r* = −0.294, *P* = 0.003).

**Figure 1:**
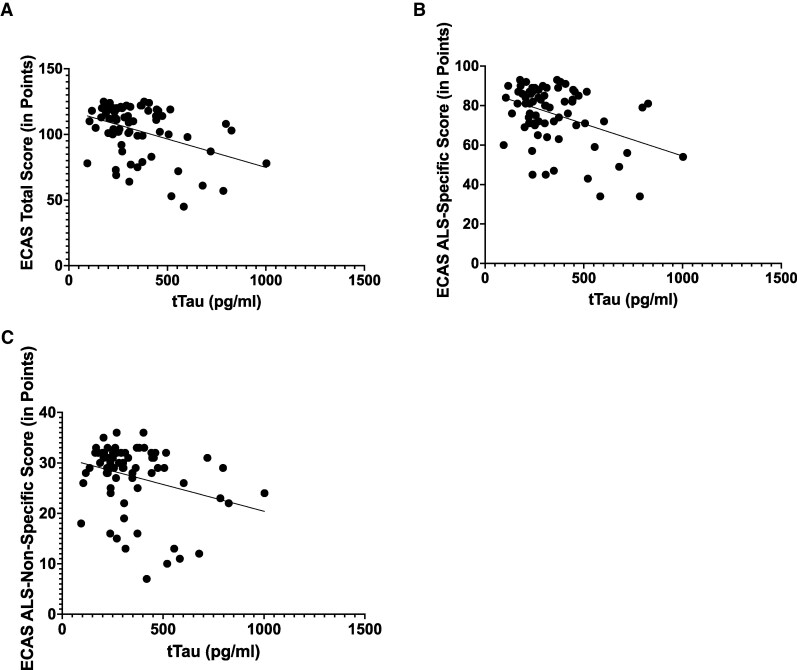
**The dependence of ECAS sub-scales on the total-tau level measured by Pearson correlation.** (**A**) The dependence of ECAS total score (max. 136 points, cut score 105) on the total-tau level (*n* = 73 patients with amyotrophic lateral sclerosis, *P*-value = 0.0004, Pearson correlation coefficient (*r*) = −0.398, Level of significance (*t*) = 3.653). (**B**) The dependence of ECAS amyotrophic lateral sclerosis-specific score (max. 100 points, cut score 77) on the total-tau level (*n* = 73 patients with amyotrophic lateral sclerosis, *P*-value = 0.0004, *r* = −0.403, *t* = 3.706). (**C**) The dependence of ECAS amyotrophic lateral sclerosis-non-specific score (max. 36 points, cut score 24) on the total-tau level (*n* = 73 patients with amyotrophic lateral sclerosis, *P*-value = 0.013, *r* = −0.288, *t* = 2.534).

In the analysis of the correlation of neurodegeneration-associated proteins with the results of the ECAS (Table 2), tTau showed a mean linear negative correlation with the ECAS total score (*r* = −0.3978, *P* = 0.00049) and the amyotrophic lateral sclerosis-specific score (*r* = −0.4026, *P* = 0.00041) of the ECAS. There was a weak linear negative correlation between tTau and the ECAS amyotrophic lateral sclerosis-non-specific score (*r* = −0.2879, *P* = 0.01349) (Fig. 1). By constructing ROC curves, we attempted to define cut-off values for tTau, considering the performance of pALS in the individual ECAS scores. With only a low AUC, no qualitatively meaningful values could be determined (AUC tTau ECAS total score = 0.336; AUC tTau ECAS amyotrophic lateral sclerosis-specific score = 0.336; AUC ECAS amyotrophic lateral sclerosis-non-specific score = 0.291). A weak linear negative correlation was found for pTau with the ECAS total score (*r* = −0.2417, *P* = 0.03937) and the ECAS amyotrophic lateral sclerosis-specific score (*r* = −0.2412, *P* = 0.03981) (Supplementary Fig. 1). Classification thresholds using the ROC curve were also unsuccessful when the AUC was too low (AUC pTau ECAS total score = 0.398; AUC pTau ECAS amyotrophic lateral sclerosis-specific score = 0.415). We were unable to demonstrate a significant correlation between pTau and the amyotrophic lateral sclerosis-non-specific score (*r* = −0.1819, *P* = 0.1234) on the basis of our data. Similarly, no significant correlations were found for amyloid beta 1–40 (ECAS total score: *r* = −0.191, *P* = 0.105; ECAS amyotrophic lateral sclerosis-specific score: *r* = −0.201, *P* = 0.088; amyotrophic lateral sclerosis-non-specific score: *r* = −0.121, *P* = 0.309) and for amyloid beta 1–42 (ECAS total score: *r* = −0.098, *P* = 0. 409; ECAS amyotrophic lateral sclerosis-specific score: *r* = −0.101, *P* = 0.393; amyotrophic lateral sclerosis-non-specific score: *r* = −0.066, *P* = 0.579) and neurofilament heavy chains (ECAS total score: *r* = −0.094, *P* = 0.424; ECAS amyotrophic lateral sclerosis-specific score: *r* = −0.102, *P* = 0.383; amyotrophic lateral sclerosis-non-specific score: *r* = −0.052, *P* = 0.656).

**Table 2:** The dependence of ECAS on different neurodegeneration-associated proteins

Epidemiological data	
Gender	
Male	48
Female	51
Average duration of illness, *n* = 95 (years)	
Average value	1.9
Standard deviation	1.9
ALSFRS-R, *n* = 83	
Average value	34.55
Standard deviation	8.29

Neurodegeneration-associated proteins (CFS)				
	*n*	*r*	*t*	*P*
ABeta40				
Total score	73	−0.191	1.640	0.105
Amyotrophic lateral sclerosis-specific score	73	−0.201	1.732	0.088
Amyotrophic lateral sclerosis-non-specific score	73	−0.121	1.024	0.309
ABeta42				
Total score	73	−0.098	0.830	0.409
Amyotrophic lateral sclerosis-specific score	73	−0.101	0.860	0.393
Amyotrophic lateral sclerosis-non-specific score	73	−0.066	0.557	0.579
Total tau				
Total score	73	−0.398	3.653	0.000
Amyotrophic lateral sclerosis-specific score	73	−0.403	3.706	0.000
Amyotrophic lateral sclerosis-non-specific score	73	−0.288	2.534	0.013
Phospho-tau				
Total score	73	−0.242	2.099	0.039
Amyotrophic lateral sclerosis-specific score	73	−0.241	2.094	0.040
Amyotrophic lateral sclerosis-non-specific score	73	−0.182	1.559	0.123
NfL				
Total score	75	−0.094	0.805	0.424
Amyotrophic lateral sclerosis-specific score	75	−0.102	0.878	0.383
Amyotrophic lateral sclerosis-non-specific score	75	−0.052	0.447	0.656

ABeta40 = amyloid beta 1–40; ABeta42 = amyloid beta 1–42; *P* = *P*-value; *r* = Pearson correlation coefficient; *t* = level of significance. The table shows the dependence of the ECAS total score, the ECAS amyotrophic lateral sclerosis-specific score and the ECAS amyotrophic lateral sclerosis-non-specific score on different neurodegeneration-associated proteins from the CSF. The negative correlation between the total tau and the results of the ECAS and the negative correlation between the phospho-tau and the ECAS total score and the ECAS amyotrophic lateral sclerosis-specific score stand out.

**Figure 2:**
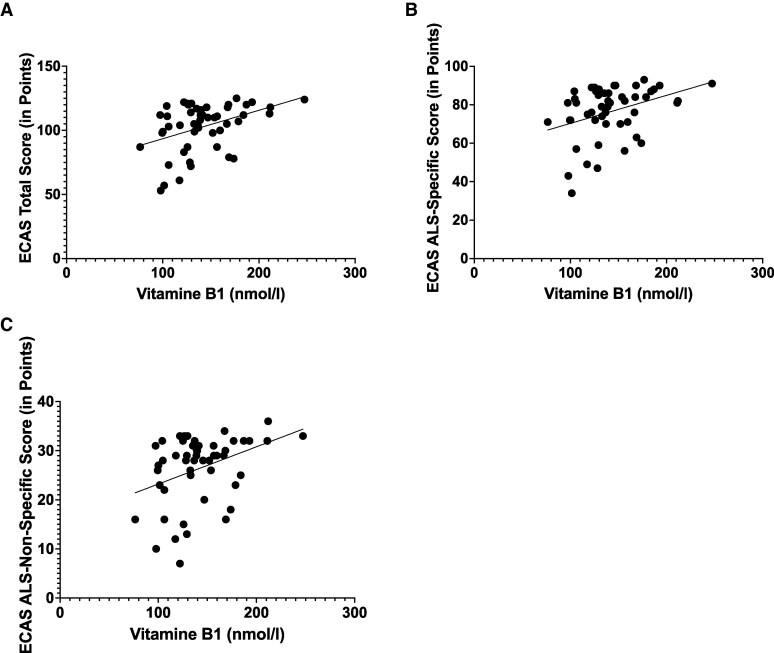
**The dependence of ECAS sub-scales on the thiamine level measured by Pearson correlation.** (**A**) The dependence of ECAS total score (max. 136 points, cut score 105) on the thiamine level (*n* = 50 patients with amyotrophic lateral sclerosis, *P*-value = 0.003, Pearson correlation coefficient (*r*) = 0.409, Level of significance (*t*) = 3.103). (**B**) The dependence of ECAS amyotrophic lateral sclerosis-specific score (max. 100 points, cut score 77) on the thiamine level (*n* = 50 patients with amyotrophic lateral sclerosis, *P*-value = 0.009, *r* = 0.365, *t* = 2.716). (**C**) The dependence of ECAS amyotrophic lateral sclerosis-non-specific score (max. 36 points, cut score 24) on the thiamine level (*n* = 50 patients with amyotrophic lateral sclerosis, *P*-value = 0.008, *r* = 0.372, *t* = 2.780).

The evaluation of the micronutrient markers (vitamin B1, vitamin B6, vitamin B12, folic acid and homocysteine) (Table 3) showed a linear correlation of medium significance between vitamin B1 and the individual ECAS scores (ECAS total score: *r* = 0.4087, *P* = 0.0032; amyotrophic lateral sclerosis-specific score: *r* = 0.3649, *P* = 0.00916; amyotrophic lateral sclerosis-non-specific score: *r* = 0.3724, *P* = 0.0077) (Fig. 2). Using ROC curves, we were able to show that a serum vitamin B1 level of less than 138 nmol/l leads to a pathological result with a specificity of 76.2% and a sensitivity of 65.5% in the ECAS total score. In the ECAS amyotrophic lateral sclerosis-specific score, pALS gives a pathological result with a specificity of 70% and a sensitivity of 60% with a serum thiamin level of up to 138 nmol/l. In the ECAS amyotrophic lateral sclerosis-non-specific score, the pALS achieved a pathological result with a specificity of 69.2% and a sensitivity of 70.3% at a Vit. B1 serum level below 129.5 nmol/l. In contrast, patients’ vitamin B12 levels showed no correlation with cognitive performance as measured by the ECAS (ECAS total score: *r* = 0.078, *P* = 0.582; ECAS amyotrophic lateral sclerosis-specific score: *r* = 0.067, *P* = 0.638; amyotrophic lateral sclerosis-non-specific score: *r* = 0.077, *P* = 0.586). This also applies to folic acid levels (ECAS total score: *r* = 0.122, *P* = 0.390; ECAS amyotrophic lateral sclerosis-specific score: *r* = 0.086, *P* = 0.544; amyotrophic lateral sclerosis-non-specific score: *r* = 0.155, *P* = 0.271), vitamin B6 levels (ECAS total score: *r* = 0.213, *P* = 0. 137; ECAS amyotrophic lateral sclerosis-specific score: *r* = 0.196, *P* = 0.171; amyotrophic lateral sclerosis-non-specific score: *r* = 0.182, *P* = 0.205) and homocysteine levels (ECAS total score: *r* = −0.191, *P* = 0.105; ECAS amyotrophic lateral sclerosis-specific score: *r* = −0.201, *P* = 0.088; amyotrophic lateral sclerosis-non-specific score: *r* = −0.121, *P* = 0.309).

**Table 3:** The dependence of ECAS on selected micronutrients

Epidemiological data	
Gender	
Male	48
Female	51
Average duration of illness, *n* = 95 (years)	
Average value	1.9
Standard deviation	1.9
ALSFRS-R, *n* = 83	
Average value	34.55
Standard deviation	8.29

Micronutrients				
	*n*	*r*	*t*	*P*
Folic acid				
Total score	52	0.122	0.867	0.390
Amyotrophic lateral sclerosis-specific score	52	0.086	0.611	0.544
Amyotrophic lateral sclerosis-non-specific score	52	0.155	1111	0272
Vit. B1				
Total score	50	0.409	3.103	0.003
Amyotrophic lateral sclerosis-specific score	50	0.365	2.716	0.009
Amyotrophic lateral sclerosis-non-specific score	50	0.372	2.780	0.008
Vit. B6				
Total score	50	0.213	1.512	0.137
Amyotrophic lateral sclerosis-specific score	50	0.196	1.388	0.171
Amyotrophic lateral sclerosis-non-specific score	50	0.182	1.284	0.205
Vit. B12				
Total score	52	0.078	0.554	0.582
Amyotrophic lateral sclerosis-specific score	52	0.067	0.473	0.638
Amyotrophic lateral sclerosis-non-specific score	52	0.077	0.548	0.586
Homocysteine				
Total score	49	−0.086	0.590	0.558
Amyotrophic lateral sclerosis-specific score	49	−0.045	0.310	0.758
Amyotrophic lateral sclerosis-non-specific score	49	−0.136	0.941	0.352

*P* = *P*-value; *r* = Pearson correlation coefficient; *t* = level of significance; Vit. B1 = vitamin B1; Vit. B6 = vitamin B6; Vit. B12 = vitamin B12. The table shows the dependence of the ECAS total score, the ECAS amyotrophic lateral sclerosis-specific score and the ECAS amyotrophic lateral sclerosis-non-specific score on the level of selected micronutrients. There is a clear positive correlation between thiamine (vitamin B1) and the ECAS scores.

Weight loss (delta BMI) of patients during the disease course up to the time of cognitive testing showed a significant negative linear correlation (Table 4). The correlation between delta BMI and the ECAS amyotrophic lateral sclerosis-specific score was weak (*r* = −0.2646, *P* = 0.0209), and the correlation between delta BMI and the ECAS total score (*r* = −0.32, *P* = 0.0048) as well as the ECAS amyotrophic lateral sclerosis-non-specific score (*r* = −0.3357, *P* = 0.00303) was moderate (Supplementary Fig. 2).

**Table 4:** The dependence of ECAS on the change of BMI in the course of the disease and the pulmonary function

Epidemiological data	
Gender	
Male	48
Female	51
Average duration of illness, *n* = 95 (years)	
Average value	1.9
Standard deviation	1.9
ALSFRS-R, *n* = 83	
Average value	34.55
Standard deviation	8.29

Change of BMI				
	*n*	*r*	*t*	*P*
BMI				
Total score	76	−0.320	0.590	0.005
Amyotrophic lateral sclerosis-specific score	76	−0.265	0.310	0.021
Amyotrophic lateral sclerosis-non-specific score	76	−0.336	0.941	0.003
**Pulmonary function**				
VCEX				
Total score	68	0.093	0.757	0.452
Amyotrophic lateral sclerosis-specific score	68	0.077	0.630	0.531
Amyotrophic lateral sclerosis-non-specific score	68	0.099	0.811	0.420

*P* = *P*-value; *r* = Pearson correlation coefficient; *t* = level of significance; VCEX = expiratory vital capacity. The first part of the table shows the dependence of the ECAS total score, the ECAS amyotrophic lateral sclerosis-specific score and the ECAS amyotrophic lateral sclerosis-non-specific score on the change in weight over the course of the disease. The change in weight over the course of the disease is described in the text as delta BMI. There is a negative correlation with all ECAS scores. The second part of the table shows that there is no correlation between the VCEX and cognitive performance as measured by the ECAS.

No relevant correlations were found for lung function, measured by VC (ECAS total score: *r* = 0.093, *P* = 0.452; ECAS amyotrophic lateral sclerosis-specific score: *r* = 0.077, *P* = 0.531; amyotrophic lateral sclerosis-non-specific score: *r* = 0.099, *P* = 0.420) and its effect on cognitive performance and behavioural abnormalities (Table 4).

Using a one-way ANOVA, we showed that the phenotypic onset of disease progression, differentiated by bulbar (*n* = 18) and spinal (*n* = 79), had no effect on ECAS scores (ECAS total score: *P*-value = 0.167; ECAS amyotrophic lateral sclerosis-specific score: *P*-value = 0.128; ECAS amyotrophic lateral sclerosis-non-specific score: *P*-value = 0.488) (Table 5).

**Table 5:** A comparison between groups: bulbar onset versus spinal onset and patients without frontotemporal dementia versus patients with frontotemporal dementia

Epidemiological data	
Gender	
Male	48
Female	51
Average duration of illness, *n* = 95 (years)	
Average value	1.9
Standard deviation	1.9
ALSFRS-R, *n* = 83	
Average value	34.55
Standard deviation	8.29

ANOVA bulbar/spinal			
ECAS total score			
	*n*	Average	
Bulbar	18	98.944	
Spinal	79	105.531	
	***F***	***P*-Value**	***F*crit**
Between groups	1.936	0.167	3.941
**ECAS amyotrophic lateral sclerosis-spec. score**			
	***n***	**Average**	
Bulbar	18	72.222	
Spinal	79	77.671	
	***F***	***P*-Value**	***F*crit**
Between groups	2.359	0.128	3.941
**ECAS amyotrophic lateral sclerosis-non-spec. score**			
	***n***	**Average**	
Bulbar	18	26.722	
Spinal	79	27.861	
	***F***	***P*-Value**	***F*crit**
Between groups	0.4857	0.488	3.941
**ANOVA frontotemporal dementia/non-frontotemporal dementia**			
**ECAS total score**			
	***n***	**Average**	
Frontotemporal dementia	6	87	
Non-frontotemporal dementia	92	105.315	
	***F***	***P*-Value**	***F*crit**
Between groups	6.038	0.016	3.94
**ECAS amyotrophic lateral sclerosis-spec.** **score**			
	***n***	**Average**	
Frontotemporal dementia	6	65.833	
Non-frontotemporal dementia	92	77.301	
	***F***	***P*-Value**	***F*crit**
Between groups	4.148	0.044	3.939
**ECAS amyotrophic lateral sclerosis-non-spec. score**			
	***n***	**Average**	
Frontotemporal dementia	6	21.167	
Non-frontotemporal dementia	92	28.108	
	***F***	***P*-Value**	***F*crit**
Between groups	7.56	0.007	3.939

*F* = *F* test; *F*crit = critical *F* value. We conclude that the onset of the disease (spinal versus bulbar) has no effect on cognitive functioning as measured by the ECAS. Our results show that the presence of frontotemporal dementia leads to poorer performance on the ECAS.

The presence of a behavioural variant frontotemporal dementia has a significant impact on ECAS scores (Table 5). In the ECAS total score, the amyotrophic lateral sclerosis-behavioural variant frontotemporal dementia patients (*n* = 6) scored significantly (*P*-value = 0.016) lower with a mean score of 87 points than the non-frontotemporal dementia-amyotrophic lateral sclerosis patients (pure amyotrophic lateral sclerosis) (*n* = 93) with a mean score of 105 points. In the amyotrophic lateral sclerosis-specific score, the amyotrophic lateral sclerosis-behavioural variant frontotemporal dementia patients achieved a mean score of 65.8 points, also significantly (*P*-value = 0.044) worse than the pure amyotrophic lateral sclerosis patients with a mean score of 77 points. The amyotrophic lateral sclerosis-behavioural variant frontotemporal dementia patients also performed significantly worse on the ECAS amyotrophic lateral sclerosis-non-specific score with a mean score of 21.1 points compared to the pure amyotrophic lateral sclerosis patients with a mean score of 28.1 points (*P*-value = 0.007).

## Discussion

### Neurodegeneration-associated proteins in cerebrospinal fluid

A lumbar puncture is a common but invasive procedure in modern medicine^18^ for the assessment of a disease of the central nervous system. It is associated with several risks. Severe side effects are rare.^19^ The determination of neurodegeneration-associated proteins is possible in a large number of laboratories in Germany and is therefore easily accessible. In the following, we outline why, despite possible inconveniences, this procedure is relevant in the diagnosis of motor neuron diseases with neuropsychological abnormalities: when evaluating diagnostic and prognostic biomarkers in CSF for cognitive performance, as measured by the ECAS in pALS, the tTau level stands out. Tau plays a key role in the intracellular transport system as a microtubule-associated protein that forms insoluble intraneuronal aggregates. Hyperphosphorylated tau no longer binds to microtubules, thus impairing transport capacity, e.g. along the axon. The tTau level shows a significant correlation, especially with the ECAS total score and the amyotrophic lateral sclerosis-specific score of the ECAS. Patients with increased tTau tended to have pathological results on the ECAS (Table 2 and Fig. 1). It can therefore be assumed that patients who are cognitively unremarkable in the context of motor symptom-dominated amyotrophic lateral sclerosis, but who have markedly elevated CSF tTau levels, carry an increased risk for an accelerated cognitive decline compared to amyotrophic lateral sclerosis cases with normal tTau levels. This suggests that tTau levels may serve as a useful diagnostic and prognostic biomarker in the diagnosis of amyotrophic lateral sclerosis. This hypothesis is supported by other studies that have investigated the correlation between tTau levels and disease progression and survival in pALS.^7,20^ Relevant correlations in terms of a worse prognosis with increased tTau were also demonstrated here. However, further longitudinal studies are needed to support this far-reaching idea. We also need further longitudinal studies to make the easily accessible tTau level even more relevant in the diagnosis of cognitive abnormalities in pALS, and to answer the question of disease progression as precisely and individually as possible. Due to recent research showing an age-dependency of tTau,^21^ we controlled for this factor in our sample to strengthen our statement about the relevance of tTau to cognition in pALS. To check the relevance of the influence of the subject's age on our results regarding the tTau value, the correlation between age and tTau was then tested, which, with an *r*-value of 0.18 and a *P*-value <0.000, showed no relevant influence of age on our tTau results. CSF phospho-tau levels also showed a significant correlation with the ECAS total score and the amyotrophic lateral sclerosis-specific score (Table 2 and Supplementary Fig. 1). We were not able to show a correlation with the amyotrophic lateral sclerosis-non-specific score in the present data. On the one hand, similar to tTau levels, we can assume that elevated pTau levels in still cognitively unremarkable pALS indicate an increased risk of early cognitive decline, and on the other hand, the pTau level gives us a tendency in which cognitive domain deficits can be expected. Longitudinal data would also be desirable for this approach. The beta-amyloids (1–40 and 1–42), which have been shown to be particularly relevant in the diagnosis of Alzheimer's disease,^22^ showed no significant correlation with cognitive performance in pALS based on our analysis. The value of CSF neurofilament heavy chains (NfL) as a marker of axonal degeneration in the central nervous system, which has been much discussed in recent years and is now relevant in the context of diagnosis,^23^ showed no significant correlation with ECAS, in line with previous observations.^6^

Currently, the possibility of determining neurodegeneration-associated proteins in less invasively accessible body fluids (blood, saliva) is being increasingly investigated.^24,25^ The measurement of neurofilament light chain in serum is already gaining acceptance in daily clinical practice and is proving to be a prognostic marker.^26^

### Nutrition

Several studies have demonstrated the importance of weight-stabilizing interventions in amyotrophic lateral sclerosis patients in relation to the progression of motor symptoms as measured by the ALSFRS-R.^27,28^ Our data analysis showed that weight loss also had a negative impact on cognitive performance as measured by ECAS (Table 3 and Supplementary Fig. 2). Unfortunately, we lack follow-up data to further substantiate this hypothesis. It remains the task of further studies to follow up on these indications. Until then, it seems advisable to counteract weight loss in pALS with regard to neuropsychological impairments.

In addition to inadequate caloric intake, for example, as a result of swallowing difficulties or changes in metabolism, a disease-adapted intake of micronutrients also plays an important role. For example, we have shown that thiamine (vitamin B1) levels, which have received little attention in amyotrophic lateral sclerosis research, appear to impact significantly cognitive performance (Fig. 2). Vitamin B1 is a water-soluble vitamin and, after absorption in the small intestine, is mainly converted to the biologically active form thiaminepyrophosphate by the enzyme thiaminepyrophosphokinase. In this form, it plays a key role in the citrate cycle. Thiamine deficiency can lead to neurodegeneration, which is clinically manifested by a triad of symptoms consisting of organic brain psychosyndrome, eye movement disorder and gait and stance ataxia (Wernicke's encephalopathy). Jesse *et al*.^29^ demonstrated thiamine deficiency in 28% of a patient population of 122 pALS. None of the patients showed the full clinical symptom triad of Wernicke's encephalopathy, but *post-mortem* histopathological criteria of Wernicke's encephalopathy were found in 2 of 23 autopsied patients. Our data show that thiamine levels in the upper normal range have a positive effect on cognition and behaviour as measured by the ECAS in all domains (Table 3). Thiamine deficiency therefore appears to be a relevant problem in pALS. According to our findings, a vitamin B1 level in the upper normal range should be aimed for in pALS. According to a review of the literature, there are indications of advantages of substitution with benfotiamine due to impaired thiamine metabolism in pALS.^30,31^ As cognitive performance secures the autonomy of pALS and overall therapeutic interventions are limited, supplementing vitamin B1 seems to represent a reasonable therapeutic strategy that should be further investigated. In addition to megaloblastic anaemia, vitamin B12 deficiency leads to hyperhomocysteinemia, peripheral polyneuropathy and, in particularly severe cases, myelopathy known as funicular myelosis. This can also lead to encephalopathy. Data published in 2022 showed a beneficial effect of high-dose methylcobalamin treatment in patients with early amyotrophic lateral sclerosis, as measured by the ALSFRS-R [12]. With regard to cognitive performance, we were unable to demonstrate a significant correlation with serum vitamin B12 levels in our patient population (*n* = 52). Nevertheless, based on the available data, we believe that it is reasonable to determine serum vitamin B12 levels and, if necessary, initiate supplementation in pALS with suppressed serum vitamin B12 levels. Our patient population is quite heterogeneous; therefore, we believe that further studies would be useful. Like vitamin B12, folic acid influences homocysteine levels. It also plays an important role in DNA nucleotide synthesis, haematopoiesis and iron metabolism. Based on our studies, we were not able to demonstrate a significant correlation between the cognitive performance of pALS and their serum folic acid levels. Data on the relevance of folic acid to cognition or disease progression/modification in pALS are scarce and more often relate to the role of folic acid in the context of homocysteine.^32^ Supplementation may be useful with regard to the influence of folic acid on homocysteine levels. We were also unable to demonstrate an association between serum vitamin B6 (pyridoxine) levels and cognition and behaviour in pALS. There is limited data on the influence of vitamin B6 levels in amyotrophic lateral sclerosis. A recently published study showed lower serum B6 levels in patients with low ALSFRS-R.^33^ In addition, vitamin B6 levels, like folic acid levels, are mainly considered modifiers of homocysteine levels. Pyridoxine acts as a catalyst in amino acid metabolism and is also involved in haematopoiesis, prostaglandin synthesis, lipid metabolism and the regulation of water balance. Regarding the now frequently mentioned homocysteine level, our data analysis showed no correlation with cognitive performance in pALS. However, a small number of studies have shown higher levels of homocysteine in pALS compared with control groups^32,34^ and, conversely, have considered the level of homocysteine to be a potential disease-modifying factor. Homocysteine is a sulphur-containing, non-proteinogenic amino acid formed as an intermediate in the breakdown of methionine. Elevated serum levels are endothelial damaging and recent data have also shown gliotoxicity.^35^ Hyperhomocysteinemia can be corrected by supplementation with vitamin B12, folic acid and vitamin B6.^36^

There is an urgent need for longitudinal studies with a personalized nutrition approach in amyotrophic lateral sclerosis, including cognitive performance and behavioural abnormalities. The weakness of our study is the retrospective data collection with only a single biomarker survey.

In view of the limited possibilities to positively influence the progression of amyotrophic lateral sclerosis, we consider the emerging options of an individually adapted high-caloric diet with monitoring of the above-mentioned micronutrients to be very positive, as they are less cost-intensive and can be influenced by the patient. Further studies, especially on the influence of vitamin B1, should be performed.

### Pulmonary function

In amyotrophic lateral sclerosis, increasing muscle weakness often leads to respiratory insufficiency with accompanying hypercapnia and consequently daytime sleepiness, which may impair concentration and attention. Several studies have shown an association between lung function and cognition.^37,38^ Like others, we analysed the VC of pALS but were not able to replicate previous observations (Table 3). One reason may be the heterogeneity of our patient population. Another possible explanation may be the challenge of measuring lung function itself. Patients need to be able to cooperate and follow instructions. Cognitively impaired patients may therefore have been excluded from our department, where lung function is assessed as an outpatient service in the pulmonary department. This cannot be reconstructed retrospectively with the available data. Ludolph *et al*. showed in 2019 that the timely use of non-invasive ventilation can stabilize and even improve the symptoms of hypercapnia.^39^

### Subgroups

In amyotrophic lateral sclerosis, two main phenotypes can be distinguished with regard to the onset of the disease—bulbar onset and spinal onset. The bulbar onset is characterized by dysarthria and dysphagia as the earliest symptoms of the disease. Spinal onset is characterized by paresis and muscle fasciculation of the extremities as the initial symptoms. In the present study, the phenotype at onset did not predict cognitive performance during the course of the disease (Table 5). It should be noted that the ECAS can be administered both verbally and in writing, thus taking into account patients’ deficits. Other researchers have also found only moderate relevance of bulbar involvement for cognitive performance in more detailed studies. Abnormalities were only found in executive functions. Nevertheless, they show a negative correlation between the extent of bulbar involvement and memory performance.^40^ However, a shortcoming of our evaluation may be the small number of patients with primarily bulbar involvement.

In amyotrophic lateral sclerosis, several neuropsychological abnormalities may occur that go beyond pathological crying or laughing. Strong *et al*.^2^ showed a high heterogeneity of amyotrophic lateral sclerosis with frontotemporal spectrum disorders. One of the aims of the ECAS is to detect behavioural abnormalities. Executive function is one of the main areas of testing. The behavioural variant frontotemporal dementia represents with particularly affected executive functions. The diagnosis of behavioural variant frontotemporal dementia is based on the Rascovsky criteria.^15^ It is therefore not surprising that patients with amyotrophic lateral sclerosis and full-blown behavioural variant frontotemporal dementia perform worse on the ECAS (Table 5). However, the deficits of patients with concomitant behavioural variant frontotemporal dementia are not limited to the scales that test executive function—pALS with concomitant behavioural variant frontotemporal dementia perform significantly worse in all domains of the ECAS based on our evaluations. This is an observation that has already been made by other researchers.^41^ A weakness of our study is the small sample size of pALS with concomitant behavioural variant frontotemporal dementia. Patients with amyotrophic lateral sclerosis and concomitant frontotemporal dementia show a more rapid loss of autonomy than pALS without frontotemporal dementia. Therefore, if pALS stands out due to highly pathological results in the ECAS, it is advisable for the clinician to discuss the prognosis and significance of amyotrophic lateral sclerosis with frontotemporal spectrum disorders with the patient and their relatives.

### Limitations of our study

Our cohort of pALS is heterogeneous. In the evaluation of the ECAS, the UK-cut scores were applied to a German population, which could result in a slight bias in the values. Given the breadth of our evaluations, we chose not to address all potential confounding factors. It is also important to note that we did not make multiple comparisons. Furthermore, there is a lack of follow-up data. Building on our findings, further focused, longitudinal studies would be beneficial.

## Supplementary Material

fcaf137_Supplementary_Data

## Data Availability

The data are available from the authors directly upon request.
