# Supplementary material for: Cognitive capacity in amyotrophic lateral sclerosis: the value of diagnostic markers in cerebrospinal fluid and the influence of nutrition and pulmonary function
Source: Brain Commun. 2025 Apr 8;7(2):fcaf137. doi: 10.1093/braincomms/fcaf137 (PMC12001800; doi:10.1093/braincomms/fcaf137)
Supplement: fcaf137_Supplementary_Data [file fcaf137_supplementary_data.pdf]

Supplemental Figures:

Cognitive capacity in amyotrophic lateral sclerosis: The value of diagnostic markers in cerebrospinal fluid and the influence of nutrition and pulmonary function

Cognition in amyotrophic lateral sclerosis

Sabrina M. Wölfel, Catherine N. Widmann, Sergio Castro-Gomez, Patrick Weydt, Pawel Tacik, Michael T. Heneka

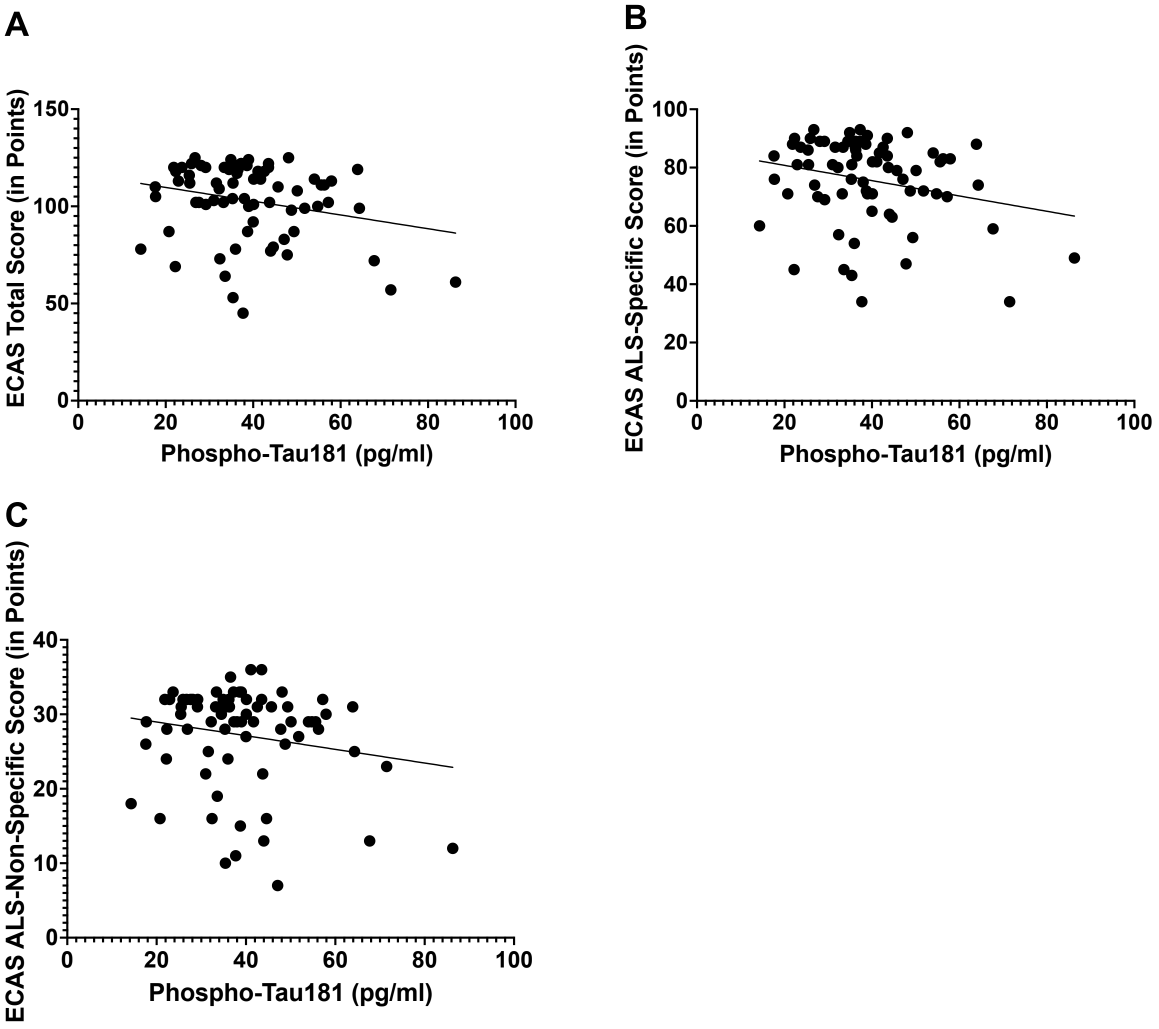

Supplementary Figure 1: The dependence of Edinburgh Cognitive and Behavioural Amyotrophic Lateral Sclerosis Screen (ECAS) sub-scales on phospho-tau181 level measured by Pearson correlation. a) The dependence of ECAS total score (max. 136 points, cut-score 105) on the phospho-Tau 181 level (n = 73 patients with Amyotrophic Lateral Sclerosis (ALS), P-value = 0.039, Pearson correlation coefficient (r) = -0.242, Level of significance (t) = 2.099). b) The dependence of ECAS ALS-specific score (max. 100 points, cut-score 77) on the phospho-Tau 181 level (n = 73 patients with ALS, P-value = 0.040, r = -0.241, t = 2.094). c) The dependence of ECAS ALS-non-specific score (max. 36 points, cut-score 24) on the phospho-Tau 181 level (n = 73 patients with ALS, P-value = 0.123, r = -0.182, t = 1.559).

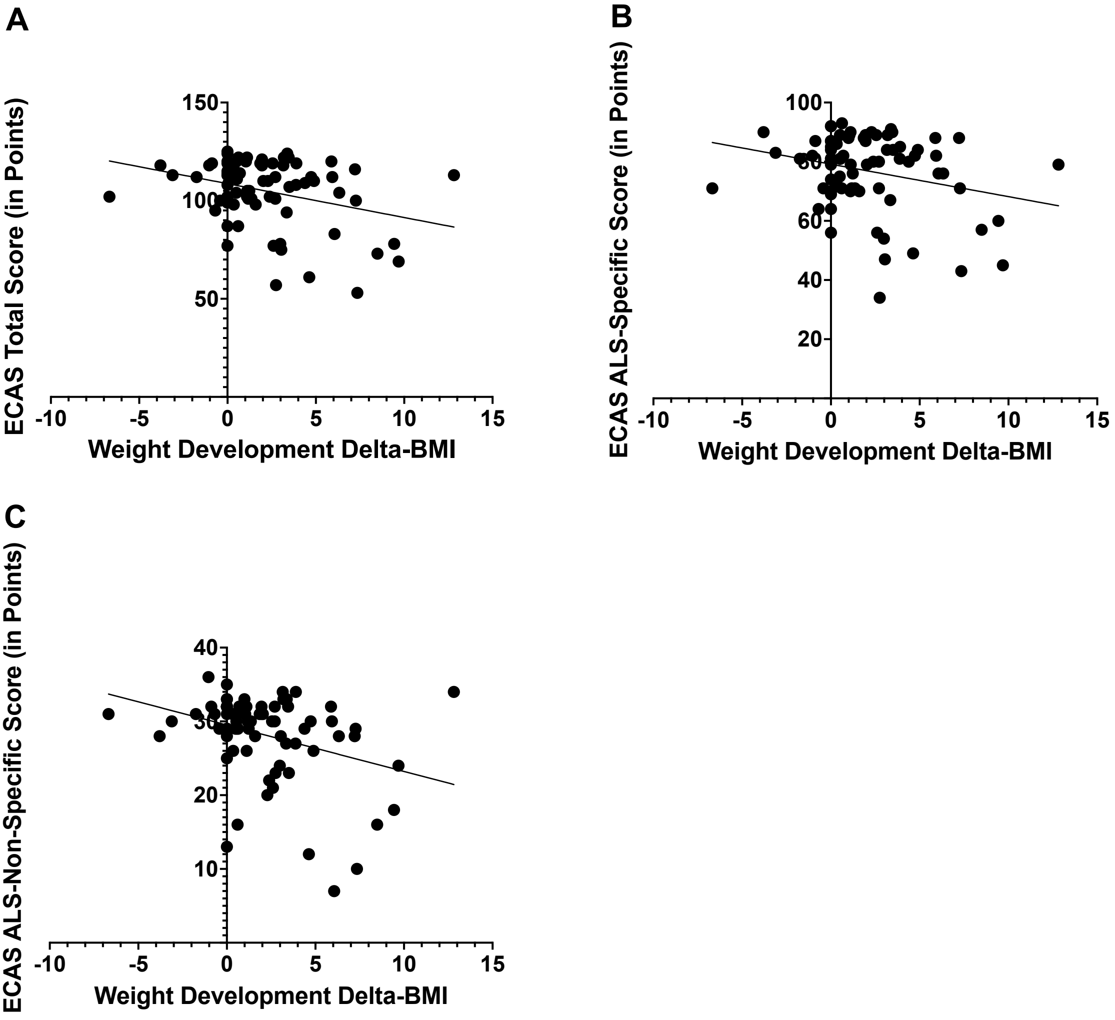

Supplementary Figure 2: The dependence of Edinburgh Cognitive and Behavioural Amyotrophic Lateral Sclerosis Screen (ECAS) sub-scales on the weight development measured by Pearson correlation. a) The dependence of ECAS total score (max. 136 points, cut-score 105) on the weight development (n = 76 patients with Amyotrophic Lateral Sclerosis (ALS), P-value = 0.005, Pearson correlation coefficient (r) = -0.320, Level of significance (t) = 0.590). b) The dependence of ECAS ALS-specific score (max. 100 points, cut-score 77) on the weight development (n = 76 patients with ALS, P-value = 0.021, r = -0.265, t = 0.310). c) The dependence of ECAS ALS-non-specific score (max. 36 points, cut-score 24) on the weight development (n = 76 patients with ALS, P-value = 0.003, r = -0.336, t = 0.941).
